# Applied improvisation and transdisciplinary simulation: a necessity for any health curriculum?

**DOI:** 10.3389/fmed.2023.1237126

**Published:** 2023-12-01

**Authors:** Julie De Wever, Mathieu Hainselin, Maxime Gignon

**Affiliations:** ^1^CRP-CPO, UR UPJV 7273, Université de Picardie Jules Verne, Amiens, France; ^2^Simulation Center, SimUSanté Epione, CHU Amiens-Picardie, Amiens, France; ^3^Department of Preventions, Risks, Medical Information and Epidemiology, CHU Amiens-Picardie, Amiens, France; ^4^Health Education and Practices Laboratory (LEPS EA3412), University Paris 13, Sorbonne Paris Cité, Bobigny, France

**Keywords:** improv, simulation, health occupations, health student, communication, decision-making, empathy

## Abstract

From practising a procedure, such as a lumbar puncture, to explaining the aim and method and listening to concerns, the practice of health professionals requires a range of skills, often classified into technical and non-technical skills. Just as gestures and procedures can be taught, so can empathy and communication skills. This article introduces an innovative approach that unites both necessary types of skills. The specific framework of improvisational theatre (“improv”) has widespread application, including the training of health professionals (health training improv). By sharing close contexts and skills, health training improv provides a valuable, safe, and effective learning environment that allows practitioners to practice exercises and situations that align with particular objectives. We created a transdisciplinary team to develop a programme of Health Professional Training Improv (HPTI), bringing together the fields of health, psychology, simulation, and arts. Since 2019, various health student groups (nurses, midwives, medical doctors, and speech therapists) have participated in a 16-h applied improv training workshop under the supervision of a professional improv facilitator. Additionally, drama students completed applied improv for health courses, which trained them to act as simulated patients, with a view to the implementation of transdisciplinary improv simulation sessions at SimUSanté (a multidisciplinary health simulation facility located in France). Students’ feedback emphasized their interest in HPTI, the realism of the simulation sessions, and the skills they felt had improved. This feedback needs to be supplemented with quantitative data from standardised assessments. The development of this rich pedagogical and research framework, based on a transdisciplinary approach, has brought different fields together to prepare students for real patient encounters. It is essential to continue this training and conduct research to evaluate the curricula developed.

## Introduction

### Background and rationale for this educational innovation

Health professionals are not machines; they are individuals who interact with others. However, future professionals are sometimes trained like robots. Skilled health professionals should have mastered specific procedures (e.g., lumbar puncture), should act quickly when a symptom appears (e.g., pain radiating from the leg), and should also be able to efficiently explain to patients the aim of an examination and listen to their concerns. Innovative training methods, based on transdisciplinarity and supported by research, are crucial, as not all health professionals receive specific training including explicit learning about communicating or empathising. This article explores how students from various health fields, including speech therapy, nursing, midwifery, and medicine, can benefit from collaboration with psychologists and improvisation actors. We examine how this collaboration helps to change their perspectives towards patients and argue for its inclusion in the health curriculum.

### Skilled health professionals and challenges in training non-technical skills

Researchers, clinicians, and educators of health professionals frequently divide medical skills into technical skills, such as performing a lumbar puncture, and non-technical skills, such as communicating information (1). This division can often lead to a hierarchy, with non-technical skills receiving less emphasis in curricula and scientific publications than technical skills (2). One might question the value of mastering cardiac resuscitation without also being able to handle time-critical decisions, or of excelling in stitching a child but faltering in reassuring them. Providing excellent care requires both technical and non-technical skills. While the usefulness of simulation training in the development of technical skills is now widely accepted (3), the type of training required to develop non-technical skills is less clear.

The belief that creativity cannot be taught, or that empathy is innate, can lead to bias in the design of curricula, and an inventive approach to learning is needed. This study examines an innovative learning approach that links crucial technical and non-technical skills. Can this approach lead to improvement in mastering them? Published studies suggest yes, and our approach also integrates expertise from other fields, enabling “thinking outside the box” (4–6).

### A New approach to train so-called non-technical skills: improvisation

Actors trained in improvisational theatre (“improv”) can use body language to express emotions, make quick decisions (e.g., in instantly choosing to behave pleasantly or to become angry in response to a health care student’s reactions), listen carefully, and adapt to the unpredictable. These skills are necessary whether they are performing on stage or working in a hospital setting. Empathic health professionals have been found to be associated with improved patient outcomes, both subjectively and objectively (7, 8). Furthermore, quick decision-making is critical because of the potential for life-threatening complications (9). However, it remains uncertain how many midwives, nurses, or other health professionals have received specific training in empathic communication or quick decision-making.

Therefore, the specific framework of improv may be a crucial component of the training of healthcare providers, and this role needs to be clarified. Recently, an increasing number of publications on this topic have emerged (4, 5, 10–18). This has led us to bring together health, psychology, and art in an unexpected but harmonious mix.

### From improvisational theatre to health professional training improv

Improvisational theatre, commonly known as “improv,” is a theatrical practice of improvised performance without a written script. Guided by core values of goodwill, mutual support, and active listening, improvisers collaborate to create stories. This embodied and integrative approach to human functioning, involving cognition, emotion, and the body in interaction with the environment, has been shown to have positive effects on participants’ memory (19), creativity (20), and tolerance of uncertainty (21). Improv techniques can have multiple applications, including in education (22), science (23), and health (13, 24). Both applied improv and emergency health care situations share common features, such as: (1) an unscripted setup with countless possibilities, (2) a dynamic environment, (3) multiple characters, (4) and an emergency that requires immediate action.

Medical improv is a form of applied improv designed for training of a variety of health professionals (24, 25), not limited to medical doctors. The more specific and inclusive term of Health Professional Training Improv (HPTI) is used here to describe such training. HPTI aims to improve health professionals’ skills, such as communication, empathy, response to time pressure, and creativity (5, 17, 24). Using the same technique as simulations, improv rehearsal provides a safe, secure, and efficient learning environment for the training of health professionals and students. Particular exercises and scenarios are selected according to the trainees’ specific needs and objectives, ensuring an optimal learning experience.

Depending on the skill being trained, HPTI facilitators have access to thousands of exercises (26, 27) and can modify them in real time during workshops. To develop empathy, health trainees can swap roles with another health improviser on stage, embodying the character and their traits. For instance, the health student playing the caregiver can become the patient, and the one playing the patient can become the caregiver, at any point during the exercise. Empathy can be conveyed (10) in various ways, including verbal, non-verbal, and paraverbal. Improv can help in improving expression of empathy by allowing individuals to embody characters in unprepared scenarios. Afterwards, constructive feedback from the audience or fellow improvisers can be provide valuable insights for improvement and enable the trainee to try again in a safe place, which is not feasible in a clinical setting with actual patients.

After each exercise, the facilitator highlights the clinical relevance of the elements addressed through debriefing. The practice of applied improvisation, similar to simulation, can be divided into several stages: (1) soliciting and enhancing the emotions of the players, (2) eliciting the emotions of the observers, and (3) providing feedback to the participants and facilitating an interactive exchange (28). While in theatrical improvisation this third phase provides feedback on the participants’ artistic performance, in applied improvisation the aim is to highlight the skills required during the exercise and draw a link to the applied discipline (28).

Thus, HPTI could, to begin with, provide an opportunity for trainees to mobilise and develop cognitive skills (perspective-taking; expression and recognition of appropriate emotions) as well as behavioral skills (verbal, non-verbal, and para-verbal communication). These skills could be applied and contextualised to clinical themes in a neutral environment, before proceeding to realistic medical environments, through simulation. Consequently, participants will be better equipped to apply these skills in real clinical contexts. The HPTI developed method is enhanced by transdisciplinary improv simulation to develop the non-technical skills of health professionals. We propose this as an innovative and viable technique to complement health professionals’ skill sets.

## Method

### The transdisciplinary educational team

We formed a transdisciplinary team in order to create the HPTI programme and associated simulations, working alongside the mental health association for health students, as well as the Theatre, Psychology, and Health departments, an experienced improv facilitator, and staff from the medical simulation centre. Transdisciplinarity “integrates the natural, social and health sciences in a humanities context, and in so doing transcends each of their traditional boundaries” and corresponds to an integrative and holistic approach which aims to integrate expertise, knowledge, and methods from the different team members and to set goals in a participatory manner (29). We prefer the term ‘transdisciplinary’ over ‘multidisciplinary’ (under which the knowledge of the various disciplines remains within the limits of their fields) or ‘interdisciplinary’ (which relates to the study and identification of links between disciplines), because this work gathered the social and health sciences in a human science context, extending traditional boundaries, which corresponds more closely to the definition of ‘transdisciplinary’ (29). Indeed, the topics of empathy and communication are not specific to any one discipline, which supports transdisciplinary work. The transdisciplinary team facilitated the amalgamation of various skill sets: medical proficiency, to ensure the clinical accuracy and pertinence of the scenarios; theatre proficiency, to instruct the actors and mentor them during the simulations; and psychological proficiency, to enhance communication and empathy in the development of scenarios and during debriefing.

The transdisciplinary team, which also represents a form of interprofessional education, convened prior to the development of HPTI and simulation training to establish the target objectives. Members of the health students’ association and medical professionals presented challenges frequently encountered by health students during their internships. Additionally, the psychologists (who are also researchers) provided guidance on emotional and relational issues and implemented the research protocol. The professional improv facilitator devised the improv training based on the objectives and needs identified.

For the second set of transdisciplinary improv simulations, the members of the health students’ association and medical practitioners collaborated to devise scenarios based on real-life situations encountered by health students. The medical practitioner attended to medical coherence and symptoms in the scriptwriting and provided medical guidance during simulation debriefings. The psychologists also contributed to the design of scenarios and debriefing, providing advice on emotional and relational issues, and oversaw the research protocol. Prior to the simulation sessions, the improv facilitator trained the simulated patients (drama students) and provided feedback on body language during debriefings. The simulation centre staff prepared the simulated environment from a technical and material viewpoint.

### The HPTI learning environment: transdisciplinary health student participants

Over the last four years, including during the COVID-19 pandemic, 63 health students (in their 2nd to 5th years of study) from various disciplines, including medicine and speech therapy, participated in a 16-h HPTI workshop, led by a professional improv facilitator. This workshop was offered as an optional course. During the HPTI workshop, the health students engaged in exercises such as the “yes and.” Professionals can employ this technique in clinical settings, for example to acknowledge a patient’s feelings during a panic attack (acknowledge with the “yes”) and collaborate to plan medical follow-up (build on it with the “and”). Additionally, they received training on using an appropriate tone of voice and maintaining professionalism in their body language, a skill that many of them needed to learn for the first time.

To prepare for the simulation sessions, third- to fifth-year drama students from the arts department underwent 12 h of training in applied improv to portray patients. This training was conducted through a separate workshop from the HPTI. The students worked on the patients’ backgrounds by improvising situations such as marital problems and were trained to realistically portray symptoms, including panic attacks. The comprehensive HPTI programme and simulation details can be accessed on the Open Science Framework (OSF) website at https://doi.org/10.17605/OSF.IO/J8WUC. The use of standardised patients entails embodiment by actors of patients executing the same actions; standardised patients are prevalent in simulations, particularly during an evaluation process. In contrast, simulated patients, who are authentic and adaptable, are beneficial for training purposes, as they enable infinite variations on the same scenario with pedagogical significance. The use of improvisational actors to portray simulated patients represents a novel approach that addresses an important need. While such actors convincingly manifest realistic symptoms and emotions, their unique preparation allows for heightened flexibility and the ability to adjust mood and language in response to live interactions.

### Transdisciplinary improv simulations

Beyond HPTI, health students were provided with the opportunity to attend non-mandatory simulated scenarios in which they assumed a caregiving role, while drama students acted as patients. Ultimately, 15% of the health students who previously trained with the HPTI programme have volunteered to be part of the improvisation simulations (more could be involved). We plan to make the simulation sessions mandatory for all HPTI-trained health students, as the culmination of their participation in their chosen optional course, and even for all health students. We carefully scheduled simulation days months in advance, taking into consideration exams and internships so as not to hinder the students’ academic progress. It may be feasible to expand the interdisciplinary team in the future by means of recruitment of additional colleagues or an increase in the available time for the existing unit, thus accommodating more students. The simulations were conducted at SimUSanté, a European multidisciplinary health simulation facility where health students are trained in both technical and non-technical skills. Health professionals are trained to manage a patient’s panic attack and draw blood samples within the same building, on the university hospital campus.

The simulation scenarios enabled students to confront clinical situations involving relational issues (e.g., the concerns of a stroke patient’s wife; the anxiety of a pregnant patient awaiting further investigations) and did not require technical actions. The documentation for each scenario included: (1) a section for the facilitators, listing the required materials, the environment, the number of arts and health students, and the possible health speciality or learning outcomes; (2) a section for the health students, providing background information and theoretical knowledge that may be useful for the situation; (3) a section for the arts students, outlining the family and professional background of their character, which they were encouraged to use to improvise in the given setting. The authors designed two versions of the scenarios by dividing the documentation into specific sections. Section 2 pertained to the health students, while section 3 was designed for the drama students. The drama students were introduced to the scenarios during their applied improv training, whereas the health students received their introduction to each scenario a few minutes before the corresponding simulation.

**Figure 1:**
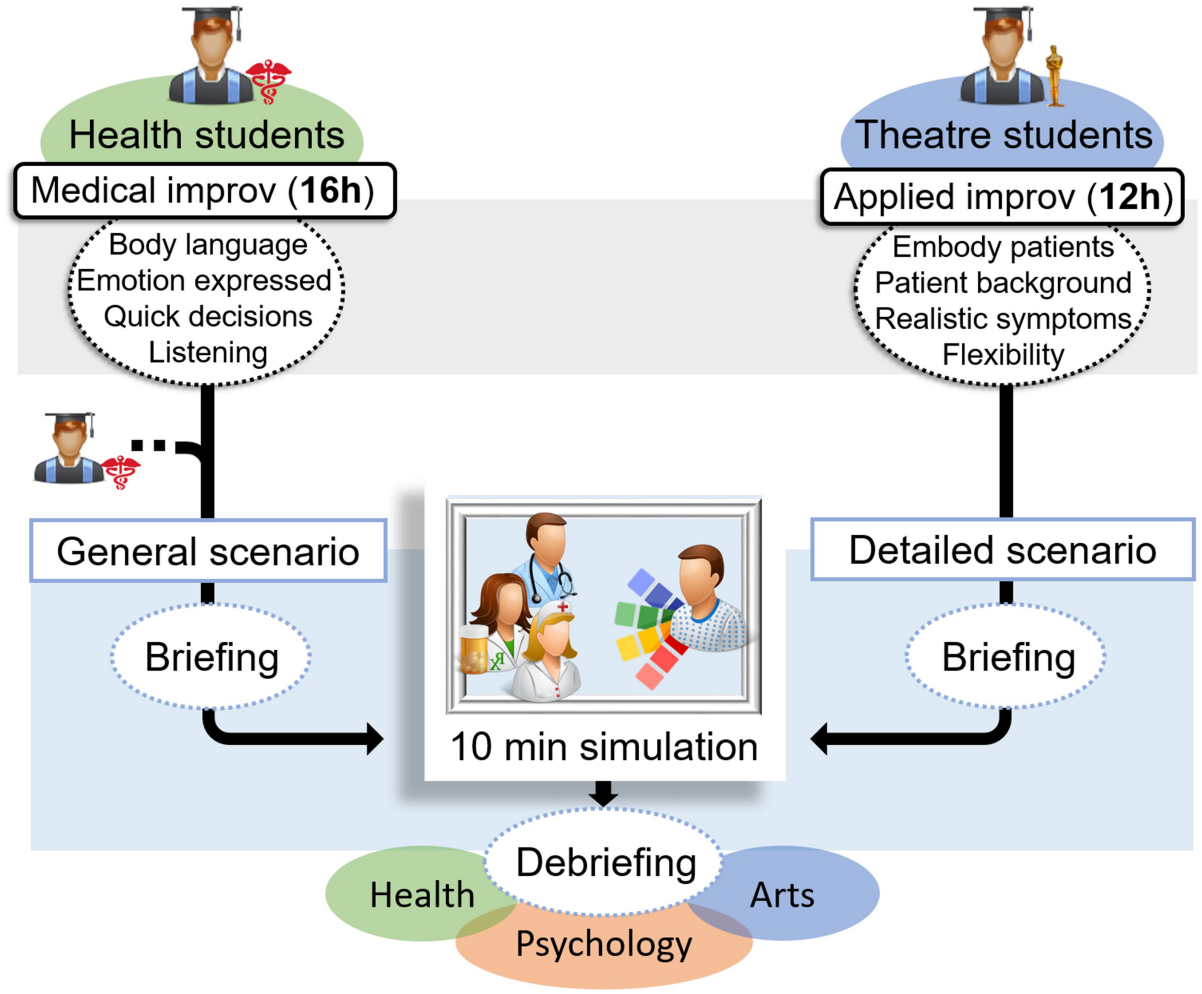
Improv Training and Simulation Organisation.

**Figure 2:**
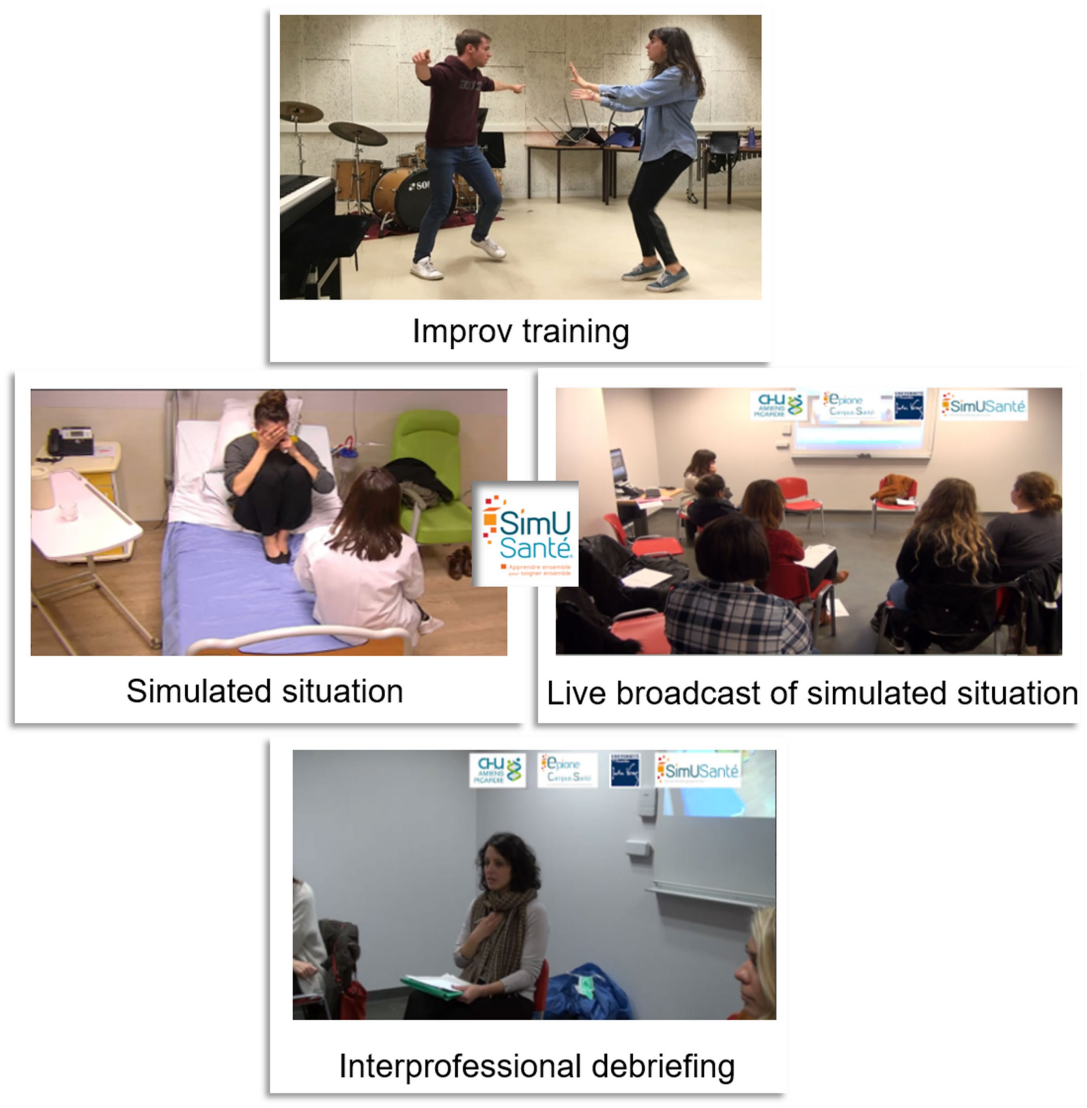
Health professional improv training and transdisciplinary simulation illustration.

On the day of the simulation session, the objectives and rules were introduced during a briefing. Before each simulation, facilitators presented the scenario to the health students. Subsequently, depending on the script, one or two health students (e.g., a health professional and their colleague) joined one or two drama students (e.g., a patient with a family member as their accompanying person) in the simulation. The simulation occurred in a simulated hospital room and was broadcast live to other students, professors, and staff in a separate room. The duration of each simulation was between 5 and 10 min, and the facilitators ended the simulation once its primary objective had been achieved. Following each simulation, several facilitators, comprising at least one professional from each field (health, psychology, and arts), conducted debriefings (Figures 1, 2). Each debriefing was based on the impressions and feelings of the health and drama students who participated in the simulation, followed by those of the observers. The students were then encouraged to identify and improve on positive aspects of the simulation collaboratively, and to work together to find ways of improving other aspects. Depending on the issues raised (medical, interpersonal, communication-related), each facilitator provided advice, additional information, and leads based on their field of expertise. As the simulated patients were portrayed by arts students who engaged in improvisation, each simulation was a new experience within the same scenario, a feature that holds pedagogical value. Each simulation introduced new behaviors to observe and debrief on, providing opportunities to try, explore, and discuss a wide range of clinical situations.

From the improv training to the simulation, both health and drama students enjoyed a secure setting, with the opportunity to halt proceedings by signalling (either verbally or via an agreed-upon gesture) to the live broadcasting camera. The only instance in which a simulation was interrupted involved a student who had encountered a creative dead end.

### Evaluation

Since 2019, eleven half-day simulation sessions have been conducted, and the sessions have been refined over time. Each session has comprised five different scenarios and has included groups of three to eight drama students and nine to seventeen health students from various fields, such as medicine, speech therapy, midwifery, and nursing care. We employed a post-improv satisfaction questionnaire and a post-simulation questionnaire to collect the health students’ impressions of the improvisation training and simulation session and improve them on this basis (example questions from the post-improv questionnaire: “Do you think that attending more similar improvisation workshops would be beneficial for your professional life?” example question from the post-simulation questionnaire: “In your opinion, what is the advantage of involving improvisational actors rather than colleagues?”). The satisfaction questionnaire was initially (before the pandemic) administered on paper, immediately following the improvisation training and the simulation session. After the pandemic, the questionnaire was administered online a few hours later and included open-ended questions and a 10-point Likert scale. This satisfaction questionnaire was part of a larger protocol that also assessed communication, empathy, and decision-making skills.

## Results: students’ feedback

To date, 61 respondent health students in their 2nd to 5th year of study in medicine and speech therapy have reported having both a professional and a personal interest in HPTI. One second-year medical student stated that “humanity plays a critical role in the medical field, and undergoing improv training provides us with a novel outlook that aids us in presenting innovative idea to enhance our patient care practices.” Students found the HPTI workshops particularly valuable for empathic communication skills. According to the feedback received, this training helped them “to put ourselves in the patient’s shoes” (comment by a second-year medical student) and “to control [my] own emotions […], to react quickly and effectively without breaking the bond of trust” (comment by a fourth-year speech therapy student). Most believed they could apply improv concepts and principles when interacting with patients or relatives, regardless of the situation or circumstances: “On the whole, it is suitable for a wide range of individuals; however it is particularly relevant for those with psychological co-morbidities or young individuals who suffer from ADHD” (comment by a fourth-year speech therapy student); “I come here and do this non-mandatory course because I really think medical professionals treat too many symptoms and not enough human, unique people with their own humanity. I think that knowing how to adapt to each person’s individuality is something we need to do, regardless of their illness or reason for consultation” (comment by a third-year medical student).

Feedback from the 93 health students (in their 2nd to 5th year of study, studying medicine, speech therapy, and pharmacy) who attended the simulation session highlighted the emotional and clinical realism of the simulation: “It would not have been the same if it had involved two health professionals or colleagues” (comment by a second-year medical student). They highlighted the particular value of using actors trained in improvisation in contrast to both their peers and non-improvising actors. A third-year pharmacy student commented: “Improvising actors are essential because they can simulate real-life scenarios with remarkable similarity.” They also valued the transdisciplinary nature of the approach and found it insightful to engage with medical students and their perspectives: “Being surrounded by medical students was interesting to see their points of view. What I really like about the simulations is debating afterwards, all the debriefings that are done afterwards, being able to discuss our postures, our ways of acting, it’s very interesting” (comment by a third-year nursing student). They also believed that their communication and empathy skills had been enhanced through participation in simulations, such as by observing simulations.

## Discussion: what to do now?

This innovative transdisciplinary programme prepares future healthcare professionals for real-life patient encounters. To our knowledge, we are among the first to combine the fields of health, psychology, and art in an improvisation and simulation curriculum. The most important lesson is the development of a very rich pedagogical and research framework, based on this transdisciplinary approach. Improvisation is one of the several tools used to enable transdisciplinary collaboration during the programme.

The HPTI training and simulation sessions were non-mandatory, which means that the positive feedback from health students should be viewed in this context. This could have led to a motivational bias (30). We can assume that all participants who chose to take part in simulation sessions placed importance on their communication skills. To overcome this limitation, it would be worth considering including these training sessions as standard practice for health students in their curricula.

During the final session break, students from the speech therapy and medicine courses engaged in a discussion regarding their respective professions, the potential for interaction, and the essential knowledge required of one another. Although this discussion took place after a debriefing session and was unscripted, we think this anecdote is another story that reinforces the impact of this pedagogical framework. Using simulation sessions through interprofessional education is a viable method of facilitating communication and the acquisition of interdisciplinary knowledge among students from various healthcare fields. While this is already common for technical activities (e.g., simulated surgery with a surgeon, nurse, etc.), transprofessional communication simulation workshops need to be more common and need to be evaluated. This article shows that it is possible to bring together health students from different backgrounds and foster their interest in learning together and learning to work together. The presence of arts students in the simulation is also a way to facilitate interdisciplinary simulation; such students bring a very different perspective because they have no specific medical knowledge and are focused on the communication component. The enhancement of the improv sessions that we have previously carried out should continue with the administration of highly complex group scenarios, such as emergency room coordination, and the evaluation of curricula through research.

To enhance the training of healthcare professionals, we strongly recommend that educators worldwide incorporate improv actors and psychologists into the curriculum, cross-training across disciplines (including nurses, midwives, physicians, speech therapists, and pharmacists) (31). These professionals will all learn to communicate together through improv and over time. This type of training is essential because we are diverse and we need every single strength to produce skilled health professionals, not robots.

## Data availability statement

The original contributions presented in the study are included in the article/supplementary material, further inquiries can be directed to the corresponding author.

## Ethics statement

The requirement of ethical approval was waived by CERNI Université de Picardie Jules Verne, Amiens, France for the studies involving humans because CERNI Université de Picardie Jules Verne, Amiens, France. The studies were conducted in accordance with the local legislation and institutional requirements. The participants provided their written informed consent to participate in this study. Written informed consent was obtained from the individual(s) for the publication of any identifiable images or data included in this article.

## Author contributions

JW wrote the article. MH and MG contributed to the writing of the article and to its review. All authors contributed to the article and approved the submitted version.
